# Validation of a screening score model to predict the development of retinopathy of prematurity

**DOI:** 10.1038/s41598-025-24303-1

**Published:** 2025-11-18

**Authors:** Johanes E. Siswanto, Asri C. Adisasmita, Sudarto Ronoatmodjo, Boromeus A. Daniswara, Peter H. Dijk, Arend F. Bos, Pieter J.J. Sauer

**Affiliations:** 1 Neonatology Working Group, Department of Pediatrics, Harapan Kita National Women and Children Health Centre, Jakarta, Indonesia; 2 Faculty of Medicine, Pelita Harapan University, Tangerang, Indonesia; 3 Department of Epidemiology, University of Indonesia School of Public Health, Depok, Indonesia; 4 Faculty of Medicine, Gajah Mada University, Yogyakarta, Special Region Yogyakarta Indonesia; 5 Department of Pediatrics, Beatrix Children’s Hospital, University Medical Centre Groningen, University of Groningen, Groningen, The Netherlands

**Keywords:** Retinopathy of prematurity, Screening model, Risk prediction, FiO₂, SpO₂, Diagnostic accuracy, Diseases, Health care, Medical research, Risk factors

## Abstract

Retinopathy of prematurity (ROP) is a leading cause of preventable blindness in preterm infants, with a disproportionate burden in low- and middle-income countries. Screening criteria from high-income settings may not be directly applicable in these contexts. We developed and validated two pragmatic risk-based screening models using multicenter Indonesian neonatal data: Model A (FiO₂-based) and Model B (SpO₂-based). Significant predictors included intrauterine growth restriction, oxygen exposure, exchange transfusion, and socioeconomic status. Internal validation showed moderate discrimination (AUC 0.719–0.732) with sensitivities of 77–86% and specificities of 44–58%. The bedside operational score form is presented for clinical use. External validation in 163 infants (gestational age 25–37 weeks, birth weight 600–2000 g) confirmed robust performance, with the combined rule (positive if either model was positive) achieving a sensitivity 84%, a specificity 81%, positive predictive value 76%, and negative predictive value 87%. The pre-test probability of ROP was 0.42, increasing to 0.76 after a positive screen and decreasing to 0.13 after a negative result. These findings support the use of locally validated risk-based scores as a practical complement to gestational age and birth weight criteria, optimizing ROP case finding in resource-limited settings.

**Supplementary Information:**

The online version contains supplementary material available at 10.1038/s41598-025-24303-1.

## Introduction

In Indonesia—and similar LMICs contexts—constrained neonatal capacity makes system-level prevention indispensable for lowering ROP risk^1^. Retinopathy of prematurity (ROP) remains a leading cause of preventable childhood blindness worldwide, particularly among preterm, low-birth-weight infants who survive owing to advances in neonatal intensive care^2–4^. Multicenter data from Indonesia show both a rising incidence of ROP and heterogeneous neonatal care practices across hospitals^2,3^. Established risk factors—including intrauterine growth restriction, oxygen exposure, and sepsis—contribute to disease onset and progression in this setting^4^.

Accurate and timely identification of infants at risk is critical, yet current screening protocols were largely derived in high-income countries and may not translate directly to LMIC care pathways^5–7^. Criteria based solely on gestational age and birthweight have shown limited sensitivity in several LMICs cohorts, underscoring the need for tailored approaches^8–10^. Weight-gain–based prediction tools (e.g., WINROP, CO-ROP, and CHOP-ROP) have shown promise in high-income settings but typically require validation before broader use in other populations^11–14^.

The global importance of ROP is emphasized in the World Health Organization’s World Report on Vision, which notes a disproportionate burden in LMICs^15^. In high-income countries, standardized screening guidance from the American Academy of Pediatrics (AAP) and partner societies has reduced missed cases and facilitated timely intervention^16^. Nevertheless, in many LMICs—including Indonesia—a “third epidemic” of ROP is emerging, fueled by increased survival of very preterm infants amid limited capacity for comprehensive screening and follow-up^4^.

To address these gaps, we evaluated two complementary models aligned with routine practice in Indonesian neonatal units: one incorporating FiO₂-based predictors (Model A) and another using SpO₂-based predictors (Model B). Against this backdrop, we sought to validate a pragmatic, risk-based screening score developed specifically for Indonesian neonatal care units^3^.

## Materials and methods

### Study design and population

We conducted a retrospective cohort study using multicenter neonatal data from hospitals and affiliated eye clinics in Jakarta, Indonesia. To ensure independent datasets, we used two non-overlapping cohorts from different time periods, with partial overlap in institutions but complete separation of subjects. The derivation cohort consisted of 175 preterm infants enrolled between 2009 and 2014 from four neonatal intensive care units (NICUs) and three affiliated eye clinics, as previously reported in our earlier work^3,4^. For the present study, the external validation cohort comprised 163 infants admitted between 2017 and 2020 at one neonatal unit and three eye clinics, including two that also contributed to the earlier cohort. Despite some overlap in study sites, the timeframes were distinct, and no infant was included in both datasets.

Infants were eligible if they had a birth weight (BW) ≤ 1500 g or a gestational age (GA) ≤ 32 weeks. In addition, infants with higher BW/GA were included if they underwent multiple ROP screening examinations and were exposed to > 40% oxygen for an extended duration during hospitalization due to cardiorespiratory instability. This definition is consistent with prior Indonesian protocols and international criteria for identifying high-risk infants^2–4^. Exclusion criteria were major congenital anomalies, incomplete medical records, or lack of documented ophthalmologic assessment.

### Ophthalmologic assessment and outcome definition

All infants were examined by pediatric ophthalmologists using indirect ophthalmoscopy after pharmacologic mydriasis. The diagnosis and staging of ROP were determined according to the International Classification of Retinopathy of Prematurity (ICROP, 2005 revision)^9^. For analytical purposes, outcomes were further categorized using the Early Treatment of ROP (ETROP) criteria into Type 1 (treatment-requiring, severe) and Type 2 (non–treatment-requiring, mild), consistent with our prior risk scoring study in Indonesian cohorts^3^. Ophthalmologists’ diagnosis served as the gold standard for model development and validation.

### Candidate predictors

Potential predictors were identified from prior literature, including Indonesian studies^2–4^, and through clinical expert consensus. Variables considered included perinatal characteristics (gestational age, birth weight, sex, intrauterine growth restriction, resuscitation at birth), neonatal morbidities (respiratory distress, sepsis, necrotizing enterocolitis, intraventricular hemorrhage), treatment exposures (oxygen supplementation, exchange transfusion, transfusions, parenteral nutrition), physiologic measures (FiO₂, SpO₂, duration of oxygen), and family background.

### Predictor screening and model entry

Candidate predictors were first assessed at the bivariable level, with variables showing *p* < 0.25 considered for multivariable logistic regression^5–7^. Multicollinearity was checked using variance inflation factors, and clinically redundant variables were excluded. Predictors retained in final models were selected by backward elimination (likelihood ratio test, exit *p* > 0.10).

### Socioeconomic status (SES)

SES was operationalized as a composite indicator comprising maternal and paternal education, household expenditure, household electricity capacity, family income relative to the regional minimum wage, and possession of health insurance^17–19^. Families were categorized as “low” or “higher” SES according to national cut-offs.

### Sample size considerations

Based on the “10 events per variable” rule^5^, a minimum of 136 infants was required to ensure adequate statistical power. Our external validation cohort comprised 163 infants, providing sufficient sample size for model testing.

### Model development and score construction

Two complementary multivariable logistic regression models were developed: Model A included FiO₂-related predictors, while Model B incorporated SpO₂ variables by design. Regression coefficients were transformed into simplified integer weights to create a bedside risk score form. The complete logistic equations and coefficients are provided in Supplementary Tables 1, and the operational bedside score form is shown in Table 2 and Supplementary Table 2.

### Statistical analysis

Model discrimination was assessed using the area under the receiver operating characteristic (ROC) curve (AUC)^8^. Calibration was evaluated with the Hosmer–Lemeshow goodness-of-fit test using deciles of predicted risk. Agreement between predicted and observed classifications was quantified with κ-statistics. Sensitivity, specificity, positive predictive value (PPV), negative predictive value (NPV), and likelihood ratios (LR + and LR−) were calculated with 95% confidence intervals. Clinical utility was further illustrated by estimating pre- and post-test probabilities using likelihood ratios and a Fagan nomogram^5,6^. All analyses were conducted in SPSS v26.

### Ethics

The study was approved by the Harapan Kita National Women and Children Health Centre Institutional Review Board. The need for informed consent was waived because data were obtained retrospectively from medical records and analysed in anonymized form. All methods were performed in accordance with relevant guidelines and regulations.

## Results

### Model development and internal validation

In the development cohort, 33 candidate variables were evaluated, of which 12 entered multivariable analysis (*p* < 0.25). These included birth weight, intrauterine growth restriction, sex, respiratory distress, asphyxia, exchange transfusion, duration of oxygen supplementation (categorized), mean FiO₂, highest FiO₂, type of oxygen supplementation, socioeconomic status, and maternal health status. Significant predictors (*p* < 0.05) were intrauterine growth restriction, exchange transfusion, duration of oxygen supplementation, and socioeconomic status. The overall model was significant (*p* < 0.001), with accuracy of 70.3%.

In Model B, FiO₂ was excluded by design; 32 candidate variables were assessed, and 10 entered multivariable analysis (*p* < 0.25): birth weight, intrauterine growth restriction, respiratory distress, hemodynamic disorders, intracranial hemorrhage, exchange transfusion, lowest SpO₂, socioeconomic status, maternal health, and father’s ethnicity. Significant predictors were birth weight, lowest SpO₂, low socioeconomic status, and exchange transfusion. Model B was overall significant (*p* < 0.001) with accuracy of 70.1%.

Full logistic equations and coefficients are provided in Supplementary Table 1. The final screening score and pre-specified operational cut-offs are shown in Table 2 (cut-off − 1.6 for Model A; 0.8 for Model B), with the bedside score form reproduced in Supplementary Table 2. Internally, both models demonstrated moderate discrimination (AUC 0.719–0.732) with sensitivities of 77–86% and specificities of 44–58%^8^. All performance metrics in this subsection refer to internal validation; external validation results are presented in Sect. "External validation of the new screening score model".

**Table 1:** Internal validation of ROP screening models against clinical diagnosis (gold standard).

ROP screening model		Clinical diagnosis (gold standard)									
		AUC	Se (%)	Sp (%)	PPV (%)	NPV (%)	LR +	LR -	Pre-test Prob	Post-test Prob	Kappa score
Prob. Model	Model A (FiO₂)	0.732	85	49	68	72	1.67	0.31	56	68	0.352
	Model B (SpO₂)	0.719	86	44	70	67	1.54	0.33	61	70	0.318
Screening score model	Model A (FiO₂)	0.730	77	58	70	67	1.87	038	56	70	0.365
	Model B (SpO₂)	0.719	85	46	71	68	1.59	0.31	61	71	0.337
Prob. Model	Combination FiO₂ + SpO₂ model	**−**	98	57	78	94	2.29	0.04	61	78	0.590
Screening score model	Combination FiO₂ + SpO₂ model	**−**	95	61	79	89	2,45	0.08	61	79	0.596

ROP screening model		Probabilistic model									
		AUC	Se (%)	Sp (%)	PPV (%)	NPV (%)	LR +	LR -	Pre-test Prob	Post-test Prob	Kappa score
Screening score model	Model A (FiO₂)	0.995	100	79	88	100	4.8	0	62	89	0.824
	Model B (SpO₂)	1.00	100	97	99	100	37	0	73	99	0.981
Screening score model	Combination FiO₂ + SpO₂ model		96	100	100	89	-	0.04	76	-	0.923

Note: κ (Kappa score) in Table 1 denotes per-threshold agreement; it is distinct from the combined-rule performance metrics.

**Table 2:** Simplified ROP risk scoring model for bedside application.

Model screening score calculation	Variable	Xn (choose one below)	Score	∑Score Model
Model A (FiO₂ model)	**IUGR**			Model A (x1 + x2 + x3 + x4 + x5 + x6)
	Prem – SGA	2	x1	
	Prem – AGA	0		
	**Sex**			
	Male	1.4	x2	
	Female	0		
	**Neonatal Respiratory Distress**			
	Positive	-3.1	x3	
	Negative	0		
	**Exchange Transfusion**			
	Yes	3.4	x4	
	No	0		
	**Oxygen Supplementation**			
	>16 days	3.9	x5	
	5–16 days	2		
	1-<5 days	1		
	Without supplementation	0		
	**Socio-economic**			
	Low	-2.8	x6	
	Upper-Middle	0		
Model B (SpO₂ model)	**Birth Weight**			**Model B** (x7 + x8 + x9 + x10)
	< 1000 g	8.1	x7	
	1000–1500 g	8.7		
	>1500 g	0		
	**Lowest SpO₂**			
	<85%	-9.6	x8	
	85–90%	1		
	>90%	0		
	**Socio-Economic**			
	Low	-7.1	x9	
	Upper-Middle	0		
	**Exchange Transfusion**			
	Yes	13.5	x10	
	No	0		

∑Score: Model A = Risk of ROP positive if the sum of scores ≥ − 1.6; Model B = Risk of ROP positive if the sum of scores ≥ 0.8.

Patients have an increased risk of developing ROP if the resulting total score meets one of the cut-off values.

### External validation of the new screening score model

The external validation cohort comprised 163 infants (gestational age 25–37 weeks; birth weight 600–2000 g). Of these, 122 were < 32 weeks, including 20 < 28 weeks. Using the International Classification of Retinopathy of Prematurity (ICROP 2005 revision)^9^, 68 infants were ROP-positive and 95 showed no ROP. Distribution by ROP type and place of birth is summarised in Supplementary Table 3.

Table 2 provides the operational bedside form of the screening score used in daily practice. For each infant, clinicians select the applicable predictors listed, and the item weights are summed to yield a total score for each model. Pre-specified thresholds from model development were applied unchanged in the validation: Model A (FiO₂-based) is screen-positive if score ≥ − 1.6 (negative if < − 1.6), and Model B (SpO₂-based) is screen-positive if score ≥ 0.8 (negative if < 0.8). The printable score form is reproduced in Supplementary Table 2. For transparency, Supplementary Table 4 presents 2 × 2 contingency tables comparing predictions from Model A, Model B, and their combination against the ophthalmologist’s diagnosis (gold standard).

Performance metrics are shown in Table 3. Considered individually, Model A and Model B performed lower than their combined rule. Using a simple combined rule—screen-positive if either Model A or Model B was positive—the screen achieved sensitivity 84%, specificity 81%, PPV 76%, and NPV 87% (LR + 4.4; LR − 0.2). This improves case finding while recognising that approximately 16% of true cases could still be missed.

**Table 3:** External validation of ROP screening models against clinical diagnosis (gold standard).

The study evidence	Sensitivity (%)	Specificity (%)	PPV (%)	NPV (%)	LR +	LR -
Prediction of Model A (FiO₂)	76	41	48	71	1.3	0.6
Prediction of Model B (SpO₂)	38	76	53	63	1.6	0.8
Predictions of combined A and/or B models	84	81	76	87	4.4	0.2

Note: PPV; Positive predictive value, NPV; Negative predictive value; LR + (Positive likelihood Ratio); LR - (Negative likelihood Ratio).

### Pre- and post-test probabilities based on the score model

To illustrate the clinical meaning of the external validation results, we calculated the shift in probability using likelihood ratios. The cohort pre-test probability of ROP was 0.42 (68/163; pre-test odds 0.72). Applying the likelihood ratios derived from the combined model, a positive screen increased the post-test probability to 0.76, whereas a negative screen reduced it to 0.13.

**Fig. 1:**
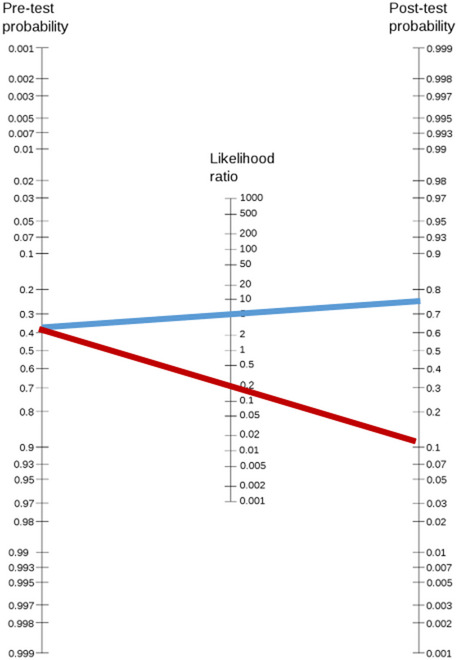
Fagan nomogram illustrating the change in ROP risk after applying the combined screening model.

Figure 1 shows the corresponding Fagan nomogram, demonstrating how the combined screening rule changes the clinician’s estimation of ROP risk in practice^5,6^. A worked example is provided: (1) Pre-test probability *p* = 0.42 ⇒ odds = p/(1 − p) = 0.42/0.58 = 0.72. (2) Positive screen: LR + = 4.4 ⇒ post-test odds = 0.72 × 4.4 = 3.17 ≈ 3.1 ⇒ probability = 3.17/(1 + 3.17) ≈ 0.76. (3) Negative screen: LR− = 0.2 ⇒ post-test odds = 0.72 × 0.2 = 0.144 ⇒ probability = 0.144/(1 + 0.144) ≈ 0.13.

Integrating the internal validation (Table 1), the operational scoring form (Table 2), and the performance in external validation (Table 3), the probability analysis underscores the model’s practical utility at the bedside and frames the discussion of how it can complement existing ROP screening strategies in LMICs settings. Evidence from external validations in middle-income populations likewise shows robust performance of simplified screening strategies, supporting generalizability beyond high-resource settings^20^.

## Discussion

Our study developed and externally validated two pragmatic screening models for retinopathy of prematurity (ROP) tailored to the Indonesian neonatal population. Both the FiO₂-based (Model A) and SpO₂-based (Model B) versions demonstrated moderate discrimination, with improved sensitivity and specificity when combined. These findings support the complementary value of physiologic variables beyond gestational age and birth weight alone, and indicate the potential of context-specific tools for use in low- and middle-income countries (LMICs)^2–4^.

Several weight-based screening algorithms have been proposed internationally^10–14^, including WINROP, CO-ROP, and CHOP-ROP, which emphasize postnatal weight gain as a key determinant of ROP risk (with similar findings reported in Brazilian very-low-birth-weight cohorts^10^. Building on this lineage, early postnatal weight-gain models such as CHOP and WINROP laid the groundwork for later refinements—G-ROP, which demonstrated high sensitivity in North American cohorts^21^, and DIGIROP, which provides dynamic risk estimates with robust external validation^22^; the updated DIGIROP 2.0 additionally incorporates parenteral-nutrition duration^23^. Collectively, these approaches highlight the diversity of available tools, yet their performance may vary in LMICs populations, underscoring the need for locally validated models.

In parallel, artificial intelligence (AI) has gained momentum as a novel approach to ROP screening. A recent systematic review synthesized the performance of multiple AI algorithms, showing encouraging but heterogeneous accuracy^24^. Pilot work has extended this concept to smartphone-based video capture with automated AI interpretation in LMICs settings^25^, and other external validations have similarly demonstrated the feasibility of AI models across diverse populations^26^. Nurse-led wide-field retinal imaging has also shown excellent accuracy in Asian cohorts, illustrating that task-shifting strategies can effectively expand screening capacity where ophthalmologists are scarce^27^. Collectively, these innovations point toward a hybrid future in which clinical scores, AI, and telemedicine complement each other to optimize screening coverage.

While scoring models provide pragmatic triage, they must be integrated with established standards of ophthalmologic evaluation^28^. Early angiographic studies have delineated vascular abnormalities that precede treatment-requiring disease^29^, while biological risk factors such as infection, oxygen exposure, and immaturity interact in the pathogenesis of ROP^30^, emphasizing the need for careful clinical follow-up. Authoritative texts such as the Moorfields Manual of Ophthalmology reinforce the role of structured examination and surveillance^31^. Thus, bedside scores should be viewed as adjunctive decision-support tools rather than replacements for specialist input.

Socioeconomic inequities also shape ROP outcomes. In the United States, disparities in access to timely screening and treatment have been well documented^18,19,32^, and broader social determinants of health—including housing stability and parental support—significantly influence outcomes^33^. Adherence to follow-up remains suboptimal, with multicenter studies reporting high attrition after discharge^32^. A systematic review of ROP guidelines highlighted marked heterogeneity worldwide, reflecting differences in neonatal care resources^16^. In LMICs, these challenges are magnified by limited infrastructure, variable oxygen monitoring practices, and scarce trained personnel. Complementary frameworks for prevention in LMICs neonatal units have therefore emphasized bundled interventions, including safe oxygen practices, nutritional support, and integrated screening pathways^15^.

**Fig. 2:**
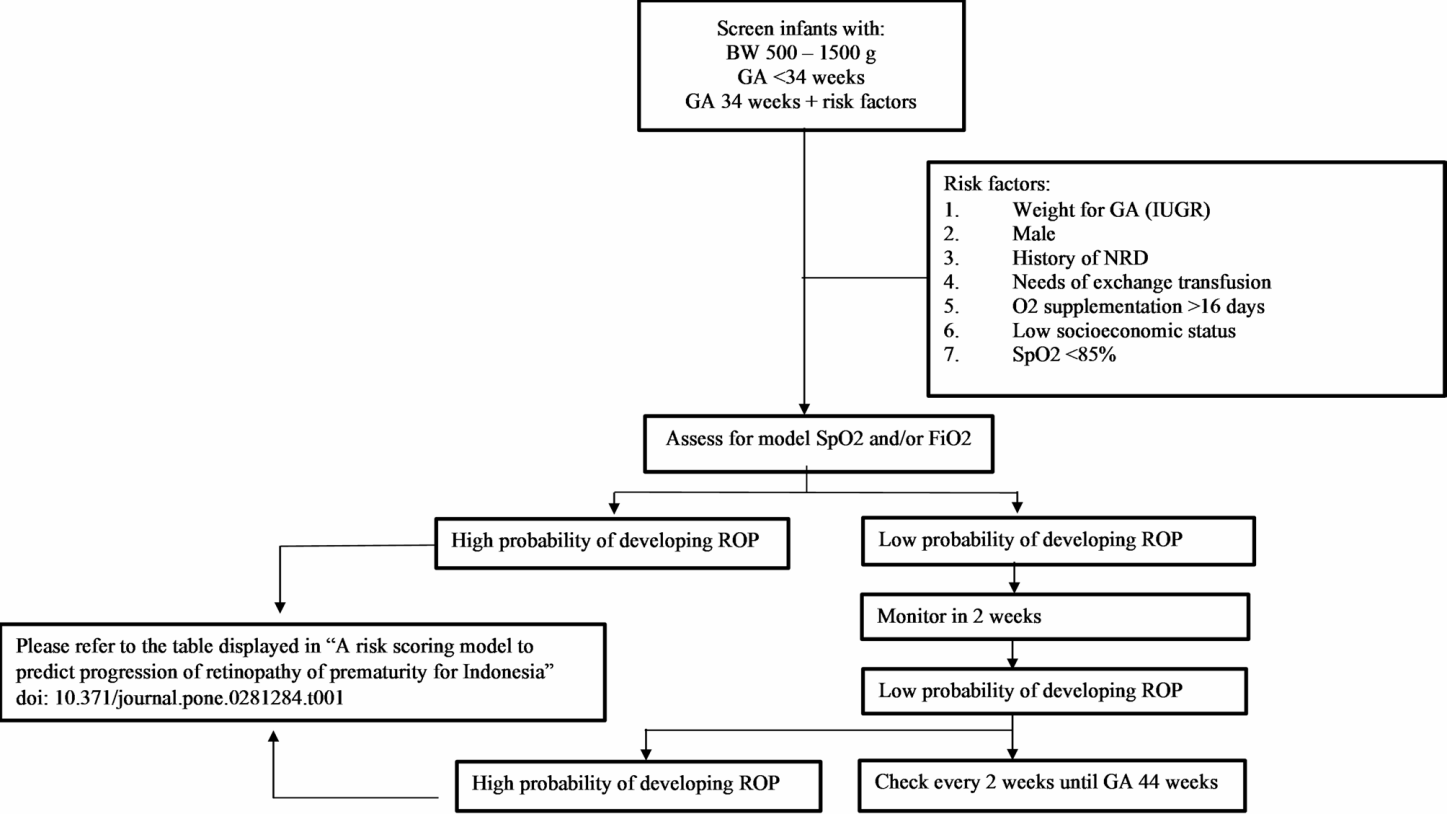
A procedural diagram designed for use by ophthalmologists or medical practitioners to evaluate the probability of an infant developing ROP.

Taken together, our findings reinforce the need for context-specific prediction tools within broader neonatal quality-improvement initiatives. International consensus guidance—such as from the AAP^16^—provides valuable benchmarks, while the WHO *World Report on Vision* underscores inequities in access^15^. Yet pragmatic, locally derived models remain essential for LMICs neonatal units to balance resource constraints with the imperative of preventing avoidable blindness. Accordingly, we provide an operational scoring form (Table 2) together with a step-by-step procedural workflow that translates the score into bedside actions (Fig. 2).

Strengths of this study include the multicenter design, incorporation of relevant clinical variables, and both internal and external validation. Limitations include the moderate sample size, reliance on retrospective data, and lack of real-time implementation testing. Nonetheless, this is the first study from Indonesia to externally validate a bedside ROP screening score that directly contrasts FiO₂- and SpO₂-based predictors. By offering an operational form ready for daily use, the score provides a pragmatic complement to existing gestational age and birth weight criteria widely applied in international guidelines^15,16^. These features highlight the novelty and practical relevance of our approach for LMICs neonatal care.

## Conclusion

We validated two pragmatic screening models for retinopathy of prematurity (ROP) in Indonesian neonatal units: one using FiO₂‑based predictors (Model A) and another using SpO₂‑based predictors (Model B). Both models showed moderate discrimination individually and improved performance in combination, incorporating locally relevant predictors such as intrauterine growth restriction, oxygen exposure, and socioeconomic status. To our knowledge, this is the first externally validated Indonesian ROP screening score that directly contrasts FiO₂‑ and SpO₂‑based approaches, reflecting routine monitoring in LMICs neonatal care. The bedside form enhances usability, and comparisons with established international models underscore the novelty and global relevance of our findings. Scaling implementation across LMICs settings—together with preventive bundles and adherence to international benchmarks—may reduce avoidable childhood blindness. In addition, low‑cost, smartphone‑based video combined with AI could further complement conventional screening workflows at scale^34^.

## Supplementary Information

Below is the link to the electronic supplementary material.

Supplementary Material 1

## Data Availability

The de-identified dataset underlying this study contains sensitive clinical information on preterm infants and is subject to institutional ethics restrictions. As such, the data are not publicly available. De-identified data and a data dictionary can be obtained from the corresponding author upon reasonable request for research purposes, contingent on approval by the relevant ethics committee and a data-use agreement.

## Abbreviations

g: grams
LMICs: low- and middle-income countries
HICs: high-income countries
ROP: retinopathy of prematurity
ROC: Receiver Operating Characteristics
AUC: area under the curve
AI: artificial intelligence
