# Supplementary material for: Validation of a screening score model to predict the development of retinopathy of prematurity
Source: Sci Rep. 2025 Nov 18;15:40571. doi: 10.1038/s41598-025-24303-1 (PMC12627429; doi:10.1038/s41598-025-24303-1)
Supplement: Supplementary file 1 — Supplementary Material 1 [file 41598_2025_24303_MOESM1_ESM.docx]

**Supplementary Table 2.** Modified score calculation for ROP screening models

| **Score Model** | **Variable** | **P-value** | **ARR (95% CI)** | β | β x 10 rounded | (β **/smallest**  **number** | **Screening**  **Model A** | **Screening Model B** |
| --- | --- | --- | --- | --- | --- | --- | --- | --- |
| **Calculation** |  |  |  |  |  |  |  |  |
| **Model A**  **(FiO₂**  **model)** | **Weight for GA** | | | | | | | |
|  | Prem – SGA | 0.037 | 2.38 (1.06-5.38) | 0.87 | 8.7 | 2 | 2 |  |
|  | Prem - AGA |  | 1.00 | - | - | - | - |  |
|  | **Gender** | | | | | | | |
|  | Male | 0.088 | 1.82 (0.92-3.63) | 0.6 | 6 | 1.4 | 1.4 |  |
|  | Female |  | 1.00 | - | - | - | - |  |
|  | **Neonatal respiratory distress** | | | | | | | |
|  | Present | 0.069 | 0.26 (0.06-1.11) | -1.33 | -13.3 | -3.1 | -3.1 |  |
|  | No |  | 1.00 | - | - | - | - |  |
|  | **Exchange Transfusion** | | | | | | | |
|  | Yes | 0.010 | 4.38 (1.42-13.55) | 1.48 | 14.8 | 3.4 | 3.4 |  |
|  | No |  | 1.00 | - | - | - | - |  |
|  | **Oxygen supplementation** | | | | | | | |
|  | >16 days | 0.001 | 5.30 (1.97-14.28) | 1.67 | 16.7 | 3.9 | 3.9 |  |
|  | 5-6 days | 0.090 | 2.36 (0.87–6.38) | 0.86 | 8.6 | 2 | 2 |  |
|  | 1-5 days | 0.420 | 1.54 (0.54-4.40) | 0.43 | 4.3 | 1 | 1 |  |
|  | Without 02 |  | 1.00 | - | - | - | - |  |
|  | **Socioeconomic status** | | | | | | | |
|  | Lower | 0.001 | 0.30 (0.15–0.63) | -1.19 | -11.9 | -2.8 | -2.8 |  |
|  | Middle – upper | - | 1.00 | - | - | - | - |  |
| **Model B**  **(SpO₂**  **model)** | **Birth Weight** | | | | | | | |
|  | < 1000 g | 0.071 | 3.38 (0.90 – 12.67) | 1.22 | 12.2 | 8.1 |  | 8.1 |
|  | 1000-1500 g | 0.023 | 3.68 (1.20-11.31) | 1.3 | 13 | 8.7 |  | 8.7 |
|  | >1500 g |  | 1.00 | - | - | - |  | - |
|  | **Lowest SpO2** | | | | | | | |
|  | <85% | 0.019 | 0.24 (0.07 – 0.79) | -1.44 | -14.4 | -9.6 |  | -9.6 |
|  | 85 - 90% | 0.876 | 1.16 (0.17 –7.77) | 0.15 | 1.5 | 1 |  | 1 |
|  | >90% | - | 1,00 | - | - | - |  | - |
|  | **Socio-Economic** | | | | | | | |
|  | Low | 0.009 | 0.34 (0.15– 0.76) | -1.07 | -10.7 | -7.1 |  | -7.1 |
|  | Middle – upper |  | 1.00 | - | - | - |  | - |
|  | **Exchange Transfusion** | | | | | | | |
|  | Yes | 0.004 | 7.55 (1.90-29.93) | 2.02 | 20.2 | 13.5 |  | 13.5 |
|  | No |  | 1.00 | - | - | - |  | - |

Note: Scores were derived by dividing each β coefficient by the smallest absolute β, then multiplying by 10 and rounding to the nearest integer.

All decimal commas were standardized to points.

# Supplementary Table 1. Logistic regression equations for ROP prediction models

| **Model** | **Logistic regression equation** |
| --- | --- |
| Model A (FiO₂) | Logit (Y \| ROP positive) = 0.45 + (IUGR × 0.87) + (Male × 0.60) – (Respiratory distress × 1.33) + (Exchange transfusion × 1.48) + (O₂ supplementation >16 days × 1.67) + (O₂ supplementation 5–16 days × 0.86) + (O₂ supplementation 1–5 days × 0.43) – (Low SES × 1.19) |
| Model B (SpO₂) | Logit (Y \| ROP positive) = 0.68 + (Birth weight <1000 g × 1.22) + (Birth weight 1000–1500 g × 1.30) – (Lowest SpO₂ <85% × 1.44) + (Lowest SpO₂ 85–90% × 0.15) – (Low SES × 1.07) + (Exchange transfusion × 2.02) |

Note: SES = socioeconomic status; IUGR = intrauterine growth restriction.

Supplementary Table 3. Characteristics of 163 infants by diagnosis of ROP and place of birth

| **Variable** | **Out-born infants (n=54)** | | | **Inborn Infants (n=109)** | | | **Total** |
| --- | --- | --- | --- | --- | --- | --- | --- |
|  | **GA <28w** | **GA 28 - 32w** | **GA >32w** | **GA <28w** | **GA 28 - 32w** | **GA >32w** |  |
| ROP positive | 9 | 21 | 3 | 5 | 21 | 9 | 68 |
| No ROP | 1 | 17 | 3 | 2 | 43 | 29 | 95 |
| Total | 10 | 38 | 6 | 7 | 64 | 38 | 163 |

Note: ROP: Retinopathy of Prematurity; GA: gestational age; w: weeks.

Out-born infants: referral cases; inborn infants: born in hospital

**Supplementary Table 4.** 2x2 table to assess the accuracy of a scoring system's diagnostic test

| **Diagnosis of ROP** | **Clinical diagnosis by an ophthalmologist** | | |
| --- | --- | --- | --- |
|  | **ROP positive (cases)** | **No ROP (cases)** | **Total (cases)** |
| **Predictive models for diagnosis** |  |  |  |
| **Model A (FiO₂)** |  |  |  |
| Risk Score ROP positive | 52 | 56 | 108 |
| Risk Score No ROP | 16 | 39 | 55 |
|  | 68 | 95 | 163 |
| **Model B (SpO₂)** |  |  |  |
| Risk Score ROP positive | 26 | 23 | 49 |
| Risk Score No ROP | 42 | 72 | 114 |
|  | 68 | 95 | 163 |
| **Combined Model A and B** |  |  |  |
| Risk Score ROP positive | 57 | 18 | 75 |
| Risk Score No ROP | 11 | 77 | 88 |
|  | 68 | 95 | 163 |

Note: True positive = risk score positive confirmed by ophthalmologist.

True negative = risk score negative with no ROP diagnosis.
